# The antiviral drug ganciclovir does not inhibit microglial proliferation and activation

**DOI:** 10.1038/srep14935

**Published:** 2015-10-08

**Authors:** Thomas Skripuletz, Laura Salinas Tejedor, Chittappen K. Prajeeth, Florian Hansmann, Chintan Chhatbar, Valeria Kucman, Ning Zhang, Barbara B. Raddatz, Claudia N. Detje, Kurt-Wolfram Sühs, Refik Pul, Viktoria Gudi, Ulrich Kalinke, Wolfgang Baumgärtner, Martin Stangel

**Affiliations:** 1 Department of Neurology, Hannover Medical School, Hannover, Germany; 2 Center for Systems Neuroscience, Hannover, Germany; 3 Department of Pathology, University of Veterinary Medicine Hannover, Hannover, Germany; 4 Institute for Experimental Infection Research, TWINCORE, Centre for Experimental and Clinical Infection Research; a joint venture between the Helmholtz Centre for Infection Research and the Hannover Medical School, Hannover, Germany

## Abstract

Ganciclovir is effective in the treatment of human infections with viruses of the *Herpesviridae* family. Beside antiviral properties, recently ganciclovir was described to inhibit microglial proliferation and disease severity of experimental autoimmune encephalomyelitis, an inflammatory model of multiple sclerosis. Microglial activation and proliferation are main characteristics of neuroinflammatory CNS diseases and inhibition of microglial functions might be beneficial in autoimmune diseases, or detrimental in infectious diseases. The objective of this study was to determine potential inhibitory effects of ganciclovir in three different murine animal models of CNS neuroinflammation in which microglia play an important role: Theiler´s murine encephalomyelitis, the cuprizone model of de- and remyelination, and the vesicular stomatitis virus encephalitis model. In addition, *in vitro* experiments with microglial cultures were performed to test the hypothesis that ganciclovir inhibits microglial proliferation. In all three animal models, neither microglial proliferation or recruitment nor disease activity was changed by ganciclovir. *In vitro* experiments confirmed that microglial proliferation was not affected by ganciclovir. In conclusion, our results show that the antiviral drug ganciclovir does not inhibit microglial activation and proliferation in the murine CNS.

Ganciclovir is currently used as an antiviral treatment for various human infections with viruses of the *Herpesviridae* family^1^,^2^. It remains the drug of choice for the prevention and treatment of cytomegalovirus (CMV) infection in transplant recipients^3^. The ability to inhibit virus replication requires intracellular phosphorylation by viral thymidine kinase to the triphosphate derivative, which is a competitive inhibitor of deoxyguanosine triphosphate thereby impairing viral DNA synthesis.

Recently, ganciclovir has been shown to have inhibitory functions on microglia and neuroinflammation in an inflammatory animal model of multiple sclerosis (MS)^4^. In murine myelin oligodendrocyte glycoprotein (MOG)-induced experimental autoimmune encephalomyelitis (EAE) ganciclovir strongly inhibited microglial proliferation, prevented T cell infiltration into the CNS, and strongly ameliorated disease severity in mice. Thus, ganciclovir was suggested as a potential novel therapeutic for neurological diseases associated with microglial proliferation and neuroinflammation.

The role of microglia in CNS neuroinflammation still remains unclear and beneficial as well as detrimental functions have been proposed. In general, activation of microglia is interpreted as a favorable inflammatory response to CNS infections where microglia derived cytokines are necessary to mount an effective immune response^5^. In CNS infectious diseases caused by herpes viruses such as CMV, inhibition of microglial functions thus might induce deleterious effects. For this reason, the aim of the present study was to determine possible inhibitory effects of ganciclovir on microglia by using three different murine models of CNS neuroinflammation in which microglia play an important role. We evaluated the effects of ganciclovir on microglia in Theiler´s murine encephalomyelitis (TME), the cuprizone model of de- and remyelination, and the vesicular stomatitis virus (VSV) encephalitis model. TME presents a virus-induced infection of the CNS and serves as an inflammation-mediated model of MS^6^. The toxic cuprizone model of de- and remyelination, in which the induction of demyelination is achieved by cuprizone feeding^7^, was used since in this model strong microglial activation occurs leading to clearance of myelin debris^8^,^9^. VSV is a rhabdovirus that causes severe and rapid encephalitis accompanied by microgliosis in mice after intranasal infection^10^. This virus has been shown to infect neuroepithelial cells, and hence is widely used as a model of acute neurotropic CNS infection^11^,^12^. The aim of our experiments was to use different animal models that each mimics different aspects of the complex mechanisms in CNS neuroinflammation. To gain further insights on inhibitory effects of ganciclovir on microglial proliferation, *in vitro* experiments with murine microglial cultures were performed.

## Results

### Microglial proliferation is a prominent feature in animal models of CNS neuroinflammation

**Figure 1:**
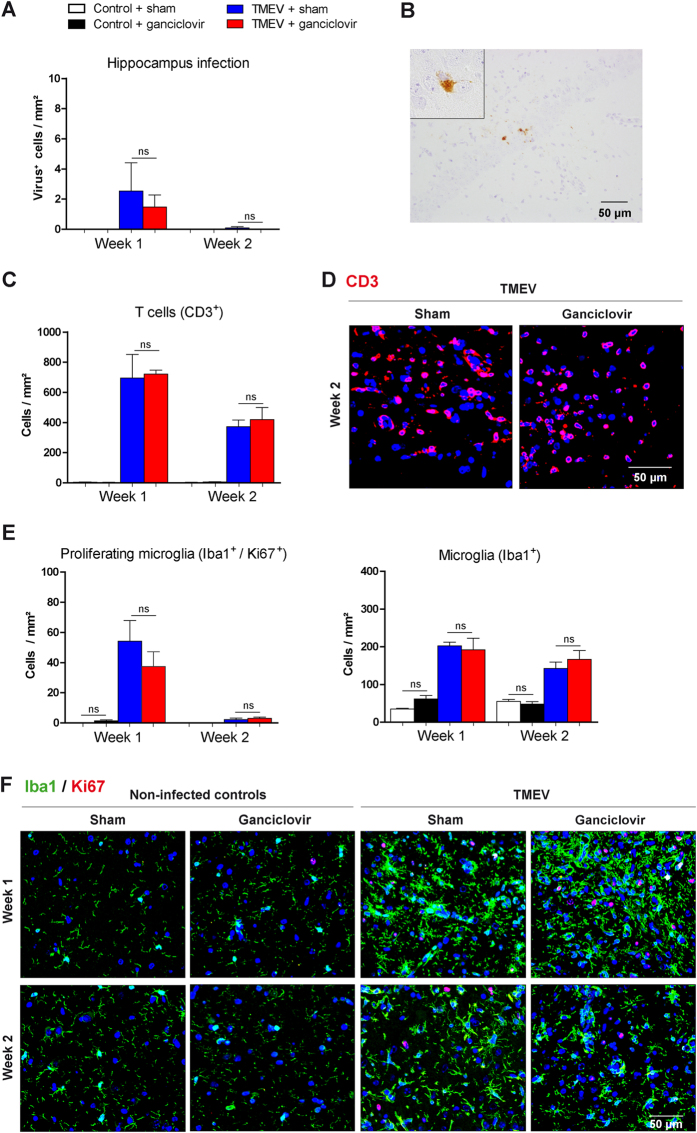
The effects of ganciclovir were investigated in Theiler’s murine encephalomyelitis virus (TMEV) infected C57BL/6 mice and in corresponding non-infected controls. Virus infection (**A**,**B**) and T cell infiltration (**C**,**D**) were confirmed by immunohistochemical stainings in the hippocampus. Total microglia numbers were analyzed by Iba1 staining, while proliferating microglia were visualized by double staining for Iba1/Ki67 (**E**,**F**). No effects of ganciclovir on virus infected neurons and T cell and microglial numbers could be observed between TMEV groups. Each bar represents the mean ± SEM, n = 6 per time point and group.

TME virus (TMEV) induced neuroinflammation is predominantly found in the hippocampus within the first weeks of infection^13^,^14^,^15^. In our experiments, TMEV infection of mice was confirmed by immunohistochemical stainings of virus particles (Fig. 1A,B) and of T cell infiltration (Fig. 1C,D) in the hippocampus and total brain (data not shown). In this model, virus clearance is fast and only few infected cells could be detected after two weeks of infection (Fig. 1A,B). We found that TMEV infection is accompanied by prominent microglial proliferation and activation (Fig. 1E,F) as also previously observed by others^16^. As compared to non-infected controls, significantly higher numbers of proliferating microglia (Iba1^+^/Ki67^+^) and total numbers of microglia (Iba1^+^) were found in the hippocampus. Proliferating microglia was detected in high numbers after one week of infection and decreased in numbers at week two.

**Figure 2:**
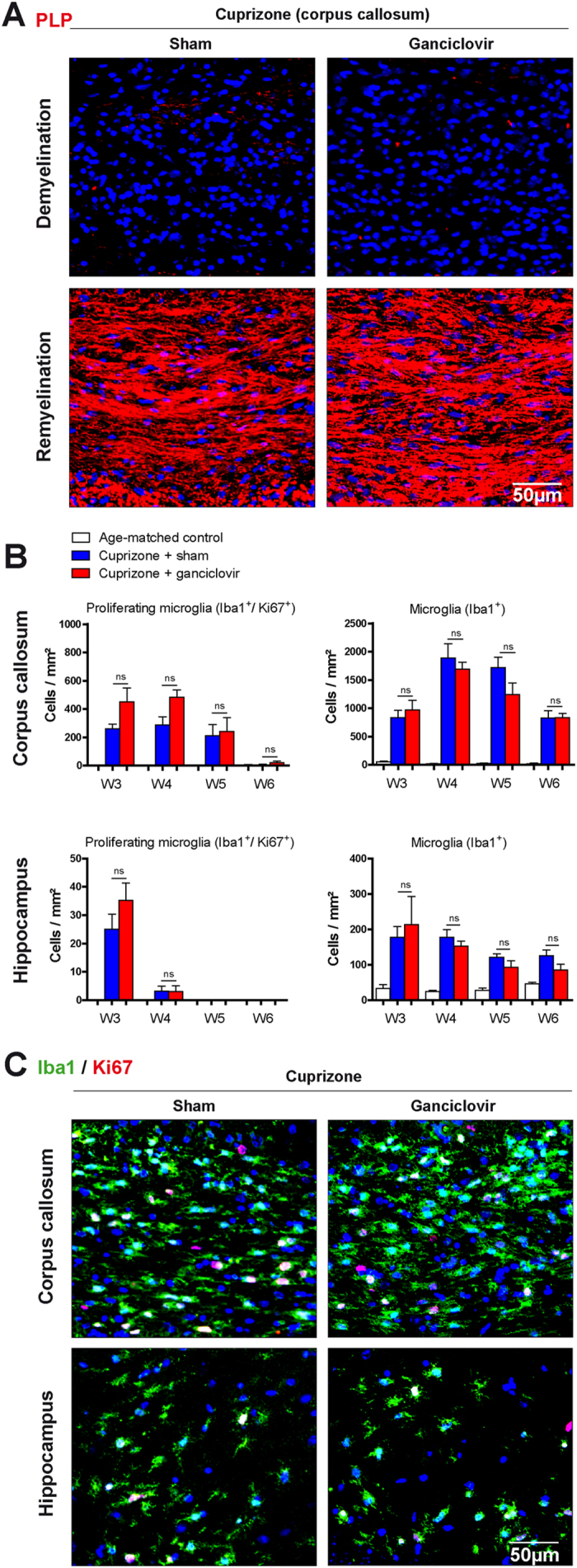
The effects of ganciclovir were investigated in the cuprizone model of de- and remyelination. After 5 weeks of cuprizone feeding, complete demyelination occurred as shown by the immunohistochemical staining for the myelin protein proteolipid protein (PLP) (**A**). One week after cuprizone withdrawal at week 6 remyelination was visible. Total microglia numbers were analyzed by Iba1 staining, while proliferating microglia were visualized by double staining for Iba1/Ki67 (**B**,**C**). No effects of ganciclovir on de- and remyelination and microglial numbers could be observed between cuprizone groups. Each bar represents the mean ± SEM, n = 6 per time point and group.

In the toxic cuprizone model of de- and remyelination, oligodendrocytes are damaged within few days of cuprizone feeding and a nearly complete loss occurs after 3–4 weeks of treatment^17^. Clearance of damaged myelin is conducted by microglial cells that are recruited towards areas of demyelination such as the corpus callosum and hippocampus^9^,^18^,^19^. To demonstrate myelin loss, brain sections were immunohistochemically stained for the myelin protein proteolipid protein (PLP). As expected, after five weeks of cuprizone treatment a nearly complete demyelination of the corpus callosum (Fig. 2A) and hippocampus (data not shown) was visible. Withdrawal of cuprizone resulted in remyelination at week 6 as shown by the re-expression of the myelin protein PLP (Fig. 2A and data not shown). Demyelination was accompanied by high numbers of proliferating microglia (Iba1^+^/Ki67^+^) and total microglia numbers (Iba1^+^) in both regions (Fig. 2B,C). The amounts and dynamics of microgliosis have been reported to be different in both regions, which might be explained by the amount of myelin to be cleared^18^,^19^,^20^. In the hippocampus, numbers of proliferating microglia (Iba1^+^/Ki67^+^) and total microglia numbers (Iba1^+^) peaked at week 3 followed by a subsequent decrease. In the corpus callosum, higher numbers of microglial cells were found and microgliosis increased up to week 4 with subsequent decrease (Fig. 2B,C).

**Figure 3:**
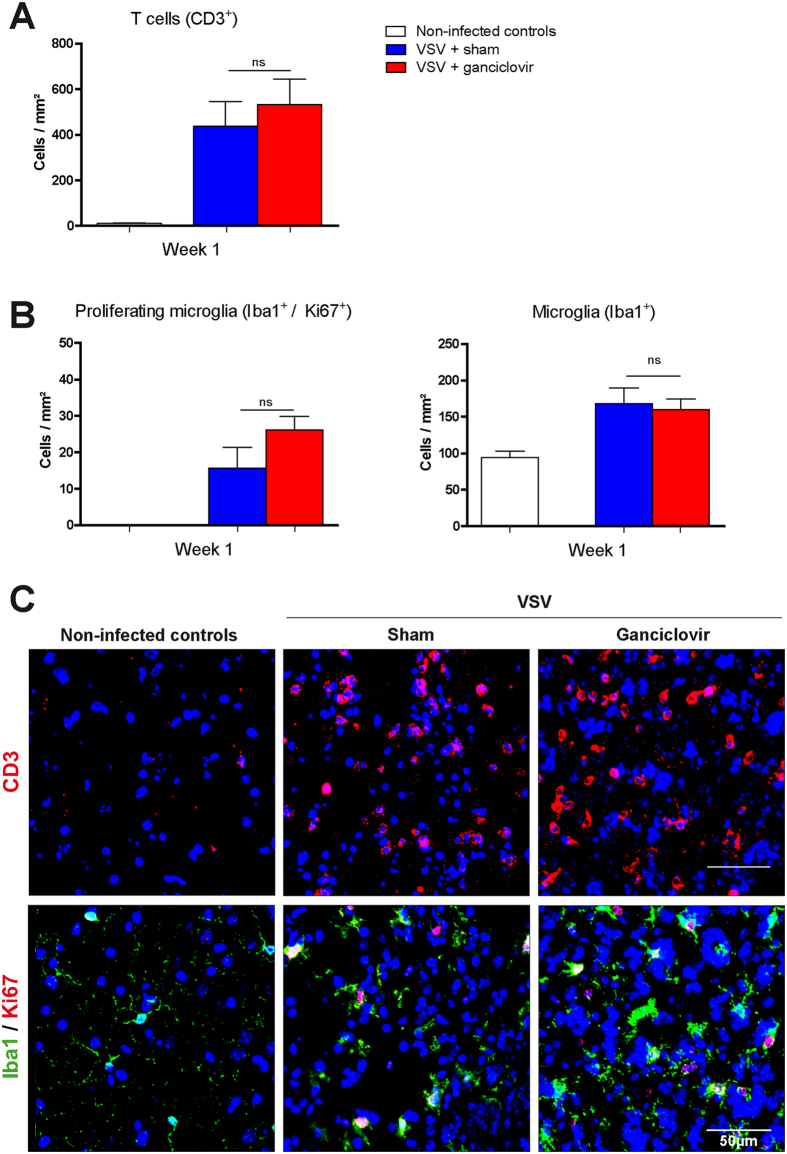
The effects of ganciclovir were investigated in the vesicular stomatitis virus (VSV) encephalitis model. Neuroinflammation was demonstrated by T cell infiltration in the cerebral cortex (**A**,**C**). Total microglia numbers were analyzed by Iba1 staining, while proliferating microglia were visualized by double staining for Iba1/Ki67 (**B**,**C**). No effects of ganciclovir on T cell infiltration and microglial numbers could be observed between groups. Each bar represents the mean ± SEM, n = 5 per time point and group.

Intranasal infection with VSV in mice results in severe encephalitis accompanied by microgliosis^10^,^12^. In our experiments, neuroinflammation was confirmed by immunohistochemical stainings for T cells in the cerebral cortex (Fig. 3A,C). After seven days of infection, an increase in the numbers of proliferating microglia (Iba1^+^/Ki67^+^) and total microglia numbers (Iba1^+^) was detectable in areas of inflammation (Fig. 3B,C).

### No effects of ganciclovir on microglial proliferation and activation *in vivo*

As shown by immunohistochemical analyses, ganciclovir did not reduce numbers of proliferating microglia (Iba1^+^/Ki67^+^) and total numbers of microglia (Iba1^+^) neither during TMEV (Fig. 1E,F), nor during cuprizone induced demyelination and the subsequent remyelination (Fig. 2B,C), nor during VSV induced encephalitis (Fig. 3B,C). In the cuprizone and VSV model, there was a tendency towards higher numbers of proliferating microglia in ganciclovir treated animals, which was not significantly changed. Additional experiments by using BrdU confirmed that ganciclovir did not change numbers of proliferating microglia (Iba1^+^/BrdU^+^) during VSV induced encephalitis (data not shown). Thus, our results show that ganciclovir did not exert any inhibitory effects on microglia numbers in three animal models of neuroinflammation.

### No effects of ganciclovir on neuroinflammation *in vivo*

Our experiments did not reveal any changes of ganciclovir on the course of CNS neuroinflammation in all three animal models analyzed in this study. In TMEV and VSV induced encephalitis, animals displayed similar T cell numbers in the brain (Figs 1C,D and 3A,C). In the cuprizone model, de- and remyelination followed the same pattern in the corpus callosum as well as in the hippocampus in both groups: ganciclovir treated animals and the corresponding controls (Fig. 2A).

### No effects of ganciclovir on proliferation and viability of primary microglia *in vitro*

**Figure 4:**
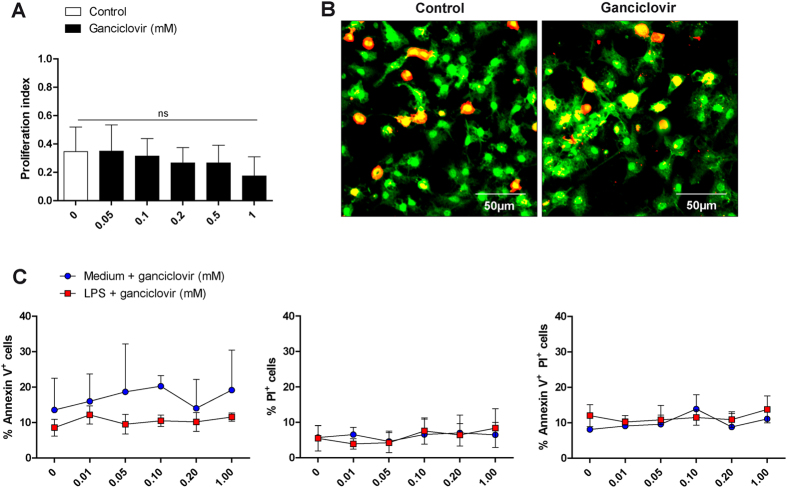
The effects of ganciclovir were investigated on the proliferation and viability of microglia *in vitro* using primary microglia cultures. Different doses of ganciclovir (0.05–1 mM) did not change microglial proliferation as shown by analysis of BrdU^+^ cells (**A,B**). The percentage of annexin V^+^ (early apoptotic), annexin V^+^ PI^+^ (late apoptotic), and PI^+^ (necrotic) cells was not changed by ganciclovir indicating that ganciclovir did not influence survival of cells. Each data represents the mean ± SEM, n = 4.

To determine whether ganciclovir might induce any cytotoxic effects on resting and activated microglia, additional *in vitro* experiments were performed. First, the effects of ganciclovir on microglial proliferation were studied. Our results show that approximately 35% of microglia harvested from neonatal brain mixed glial cultures exhibited proliferation after re-plating, as demonstrated by incorporation of BrdU into these cells. Treatment with low doses of ganciclovir (0.05–0.1 mM) for 48 h did not have any impact on the proliferation of microglia (Fig. 4). At higher doses of ganciclovir (0.2–1 mM) a small decrease in the number of BrdU^+^ cells occurred, but this reduction did not yield a statistical significance (Fig. 4A,B).

We further studied the viability of microglia harvested from neonatal brain cultures in response to increasing doses of ganciclovir (0.01–1 mM) in the presence or absence of LPS. After 48 h microglia were stained for annexin V and PI and analyzed by flow cytometry. Irrespective of the activation state of microglia, we did not observe any significant difference in the percentage of annexin V^+^ (early apoptotic), annexin V^+^ PI^+^ (late apoptotic), and PI^+^ (necrotic) cells (Fig. 4C). In further experiments we tested the effects of ganciclovir after 72 h of treatment with ganciclovir and the results were similar (data not shown). Thus, our *in vitro* findings demonstrate that treatment with ganciclovir neither affected the survival nor had any significant effects on the proliferation of primary microglia. This is in accordance with our *in vivo* findings.

## Discussion

Our experiments evaluated the effect of ganciclovir in different *in vivo* models of CNS neuroinflammation and *in vitro* cultures. Since a previous study reported the influence of ganciclovir on microglia^4^, we specifically investigated the immunosuppressive activity on this glial cell type in other models of neuroinflammation. However, we did not observe a significant difference in numbers, proliferation rates, or cellular viability of microglia. Thus, we conclude that ganciclovir is not an inhibitor of microglial activation or proliferation.

In the work of Ding *et al.* (2014), ganciclovir drastically reduced EAE disease severity. Over the course of the disease, mice in the EAE control group suffered from hind limb paralysis and forelimb paresis, while ganciclovir treated animals only displayed loss of tail tone. The large difference between groups in disease severity persisted until the end of the observation period of 21 days. Thus, the authors suggested the substance ganciclovir as a potential novel therapeutic for neurological diseases associated with microglial proliferation and neuroinflammation. Since we could not find any effects of ganciclovir in three animal models of neuroinflammation, and because the experiments were even performed in three independent labs, we conclude that the effects of ganciclovir are limited to the EAE model and will rather not play a significant role in human diseases. In addition, the ganciclovir doses that induced inhibitory effects on neuroinflammation in EAE were very high for intraperitoneal injections with 25 and 100 mg ganciclovir per kg body weight^4^. However, the parenteral doses used in humans for treatment of cytomegalovirus infection are lower^2^ and limited to a standard dose of 10 mg per day per kg body weight.

In our experiments we used a similar high dose of 25 mg ganciclovir per kg body weight in TMEV and cuprizone experiments. In VSV encephalitis we even used a higher dose of 50 mg ganciclovir per kg body weight. Since the experiments based on comparable drug doses among the EAE and our animal models, we suggest that the assessed ganciclovir effect is dose independent. The pharmacokinetics of ganciclovir in plasma and CNS were studied in animals including primates as well as in humans and it was shown that ganciclovir penetrates into the CSF in both inflammatory and resting conditions with an intact blood-brain-barrier^21^,^22^,^23^. Thus, it can be assumed that ganciclovir reached sufficient concentrations in all animal models used in our study. Furthermore, we have shown previously that the administered ganciclovir regimen is biologically active and efficient to deplete astrocytes in the cuprizone model in transgenic mice that express the herpes simplex thymidine kinase under the GFAP promoter^8^. Limitations of our study are small animal numbers and the lack of functional assays which is difficult in the three models used.

The reason for the contrary findings might be explained by the pathophysiological differences in the animal models. Peripheral immune cells and especially T cells play a key role in EAE induction and it was shown that T cells reside within the bronchus-associated lymphoid tissue of the lungs where priming takes place before entering the CNS^24^. Thus, it might be speculated that ganciclovir there exert its immune modulatory functions on immune cells that could inhibit their migration towards the CNS. Consequently, when treatment with ganciclovir was started after the EAE mice reached a high disease score of 2 corresponding to hind limb weakness or paresis, disease severity was not significantly changed^4^.

Interestingly, the effects of ganciclovir in EAE were already tested in transgenic GFAP-TK mice, in which the treatment with ganciclovir leads to loss of reactive astrocytes^25^. In this work, transgenic mice that were treated with ganciclovir exhibited a severe clinical course of EAE and higher numbers of Iba1^+^ cells in the white and grey matter of the CNS as compared to controls without ganciclovir treatment. However, the high numbers of Iba1^+^ cells are most probably a consequence of astrocyte ablation and thus the comparison of these results have some limitations. Toft-Hansen *et al.* demonstrated higher numbers of infiltrating macrophages into the CNS of ganciclovir treated GFAP-TK transgenic mice as well^26^. In this work, additional controls included wildtype C57BL/6 mice that were treated with ganciclovir or sham beginning on the first day when each mouse presented definite EAE symptoms. Treatment with ganciclovir did not show any effects on numbers of macrophages/microglia and neuroinflammation during EAE^26^.

Ganciclovir presents an important drug for the treatment of cytomegalovirus infections in humans. However, due to its high toxicity its role is rather limited in human herpes simplex and varicella zoster infections, and acyclovir is preferentially used. Even if there would be an effect on microglial proliferation and neuroinflammation in humans, we conclude that ganciclovir would not play an important role in human autoimmune disorders such as multiple sclerosis.

## Methods

### Experimental design

The objective of the study was to determine the effects of the drug ganciclovir on microglial proliferation and activation by using three different murine animal disease models of CNS neuroinflammation and *in vitro* experiments. Analyses were performed using two animal models of MS (TME and cuprizone) and a murine model of acute encephalitis induced by intranasal infection of the vesicular stomatitis virus (VSV). *In vitro* experiments were performed by using murine microglia. The ganciclovir (Cymevene, Roche) used was previously shown by us to be biologically active and efficient to deplete astrocytes in transgenic mice^8^.

### Animals

Animal experiments were performed in three independent labs (TMEV infection experiments at the University of Veterinary Medicine Hannover, cuprizone experiments at Hannover Medical School, and VSV infection experiments at the Institute for Experimental Infection Research TWINCORE Hannover). For all animals, food and water were available ad libitum. All research and animal care procedures were approved by the review board for the care of animal subjects of the district government and performed according to international guidelines on the use of laboratory animals.

### Theiler’s murine encephalomyelitis infection and ganciclovir treatment protocol

For TME, five-week-old female C57BL/6 mice (Harlan) were infected intracerebrally with 20 μl containing 1.14 × 10^5^ PFU/mouse of the BeAn strain of TMEV or mock (vehicle only) under general anesthesia using a combination of medetomidine and ketamine as previously described^6^,^27^. Animals were treated with ganciclovir (25 mg per kg) intraperitoneally or sham every day beginning on the day of infection until termination of the experiment. The four groups included (I) TMEV infected mice and treated with ganciclovir; (II) TMEV infected mice and treated with sham; (III) mock infected mice and treated with ganciclovir; (IV) mock infected mice and treated with sham. After 1 week and 2 weeks of infection mice were transcardially perfused with phosphate buffer. Brains of the four groups were removed and fixed in 4% buffered formaldehyde^6^. A group size of six animals was analysed at each time point.

### Cuprizone induced demyelination and ganciclovir treatment protocol

Experimental toxic demyelination was induced by feeding eight-week-old male C57BL/6 mice (Jackson Laboratory) a diet containing 0.2% cuprizone (bis-cyclohexanone oxaldihydrazone, Sigma-Aldrich) mixed into a ground standard rodent chow^8^. The cuprizone diet was maintained for 5 weeks. Animals were treated with ganciclovir (25 mg per kg) intraperitoneally or sham every day beginning on the day of cuprizone feeding until termination of the experiment. A group size of six animals was analysed at each time point (week 3, 4, and 5 for demyelination, week 6 for remyelination). Mice were perfused with 4% paraformaldehyde in phosphate buffer through the left cardiac ventricle and brains were removed as described^8^.

### Vesicular stomatitis virus infection and ganciclovir treatment protocol

For VSV infection, twelve-week-old female C57BL/6 mice were purchased from Harlan-Winkelmann (Borchen). Prior infection, mice were anesthetized with ketamine/xylazine and a total of 10 μl containing 10^3^ PFU of VSV in PBS were pipetted into both nostrils^11^. In order to investigate anti-inflammatory effects of ganciclovir, a group of VSV infected mice was treated with ganciclovir intraperitoneally or sham for controls every day beginning on day 1 p.i. at a dose of 50 mg/kg. On day 7, mice were perfused with 4% formaldehyde in saline and brains were removed^11^. In an additional experiment BrdU (10 μl/g, Life Technologies) was applied every day beginning on the day of VSV infection until termination on day 7.

### Immunohistochemistry

Immunohistochemistry was performed as previously described^6^,^17^,^18^,^28^. Brains were removed, postfixed in 4% PFA and paraffin embedded. For immunohistochemistry the following primary antibodies were used: for myelination the proteolipid protein (PLP) (mouse IgG, 1:500, Serotec), for microglia Ionized calcium-binding adaptor molecule 1 (Iba-1; rabbit anti-Iba-1, 1:1000, Wako), for T cells (rabbit IgG, 1:500, Dako), for proliferation Ki67 (mouse IgG, 1:50, BD Pharmingen) and BrdU (mouse IgG, 1:200, Roche diagnostics GmbH). Immunohistology targeting TMEV was performed using an anti-TMEV antibody (polyclonal rabbit anti-TMEV capsid protein VP1, 1:2000).

### Quantification of glial reactions

In TMEV infected and cuprizone treated animals brain sections between bregma −0.82 mm and bregma −1.82 mm were analysed. For VSV encephalitis experiments, brain sections between bregma +4.28 mm and bregma +3.92 mm were analysed. Quantification of glial cells was performed for proliferating microglia (double staining with Iba1/Ki67) and the total microglial numbers (Iba1). Immunopositive cells with identified nucleus (counterstaining with Mayer’s haemalum solution for immunohistochemistry or 4′,6-diamidino-2-phenylindole (DAPI) for immunofluorescence) were counted in the hippocampus of TMEV infected and cuprizone fed mice and their corresponding controls using a magnification of ×200 (Olympus BX61)^20^. In addition, in the cuprizone model cells were counted in the central part of the corpus callosum as previously described^8^. In results counted cells are expressed as number of cells per mm^2^. The extent of demyelination in the corpus callosum of cuprizone fed mice was analysed by light microscopy (Olympus BX61) as described previously^29^. For determination of demyelination in the corpus callosum PLP stained brain sections were scored in a blinded manner by three observers and graded on a scale from 0 (complete demyelination) to 3 (normal myelin).

### Microglia cell culture

Primary cultures of microglia were prepared from neonatal C57BL/6 murine brains as previously described^30^. The mixed glial cells obtained from brains were seeded into poly-L-lysine-coated T75mm^2^ culture flasks in medium consisting of DMEM + L-Glutamine +4.5 g/L D-Glucose (Gibco) supplemented with 10% fetal calf serum (FCS), 50 U/ml penicillin, and 50 μg/ml streptomycin (all Biochrom AG) and were cultured for 8–12 days with medium change on every fourth day. Once the microglia reached optimal confluency (usually between day 9–11 of culture) they were harvested by shaking the flasks at 37 °C and 180 rpm for 30 min on an orbital shaker.

### Microglia proliferation assay

Microglia proliferation was measured by the detection of 5-bromo-20-deoxyuridine (BrdU) (Roche diagnostics GmbH) incorporation into the DNA. Microglia were treated with increasing concentrations of ganciclovir (0.05–1 mM) in the presence or absence of LPS (100 ng/ml; E. coli 055:B5; Sigma-Aldrich) and incubated for 48 h. BrdU (10 μM) was added to the medium for the last 24 h. Cells were fixed with 4% paraformaldehyde and stainings for microglia (Iba1, Wako) and BrdU were performed as previously described^30^. Labelled cells were analysed using a fluorescent microscope and proliferation index was calculated by dividing number of BrdU-positive cells with total number of Iba1 positive cells.

### Annexin V and PI staining

Cell death and apoptotic cells were assessed by using FITC Annexin V apoptosis detection kit I (BD Pharmingen). Microglia harvested from mixed glial preparations as mentioned above were plated in 12-well plates at 2.5 × 10^4^ cells per well and treated with different concentrations of ganciclovir (0.01–1 mM) for 48 h. Subsequently, microglia were harvested with Trypsin-EDTA (Biochrom) and stained with FITC Annexin V and propidium iodine (PI). Staining was performed according to the instructions provided by the manufacturer. After staining, cells were analyzed on a FACS calibur (BD Biosciences)^31^.

### Statistical analysis

Statistical analysis was performed using ANOVA followed by the Fisher-protected least-significant difference test for post hoc comparison if appropriate. P values are given in the results while group comparisons derived from post hoc analysis are provided in the figures (*P < 0.05; **P < 0.01; ***P < 0.001).

## Additional Information

**How to cite this article**: Skripuletz, T. *et al.* The antiviral drug ganciclovir does not inhibit microglial proliferation and activation. *Sci. Rep.* **5**, 14935; doi: 10.1038/srep14935 (2015).
